# Sydney Melancholia Prototype Index (SMPI): translation and cross-cultural adaptation to Brazilian Portuguese

**DOI:** 10.1590/2237-6089-2019-0045

**Published:** 2020-10-08

**Authors:** Mateus Frizzo Messinger, Marco Antonio Caldieraro, Bruno Paz Mosqueiro, Felipe Bauer Pinto da Costa, Gabriela Maria Pereira Possebon, Pedro Victor de Lima Nascimento Santos, Gordon Parker, Marcelo P. Fleck

**Affiliations:** 1 Programa de Pós-Graduação em Psiquiatria e Ciências do Comportamento Universidade Federal do Rio Grande do Sul Porto AlegreRS Brazil Programa de Pós-Graduação em Psiquiatria e Ciências do Comportamento, Universidade Federal do Rio Grande do Sul (UFRGS), Porto Alegre, RS, Brazil.; 2 Hospital de Clínicas de Porto Alegre Porto AlegreRS Brazil Serviço de Psiquiatria, Hospital de Clínicas de Porto Alegre, Porto Alegre, RS, Brazil.; 3 Universidade do Vale do Rio dos Sinos São LeopoldoRS Brazil Universidade do Vale do Rio dos Sinos (UNISINOS), São Leopoldo, RS, Brazil.; 4 Black Dog Institute School of Psychiatry University of New South Wales SydneyNSW Australia Black Dog Institute, School of Psychiatry, University of New South Wales, Sydney, NSW, Australia.

**Keywords:** Depression, depression scales, melancholia, translation, adaptation

## Abstract

**Introduction:**

Depression is possibly not a single syndrome but rather comprises several subtypes. DSM-5 proposes a melancholia specifier with phenotypic characteristics that could be associated with clinical progression, biological markers or therapeutic response. The Sydney Melancholia Prototype Index (SMPI) is a prototypic scale aimed to improve the diagnosis of melancholia. So far, there is only an English version of the instrument available. The aim of this study is to describe the translation and adaptation of the English version of the SMPI into Brazilian Portuguese.

**Methods:**

Translation and cross-cultural adaptation of the self-report (SMPI-SR) and clinician-rated (SMPI-CR) versions into Brazilian Portuguese were done following recommendations of the International Society for Pharmacoeconomics and Outcomes Research (ISPOR). This guideline includes the following steps: preparation, forward translation, reconciliation, back translation, back translation review, harmonization, cognitive debriefing, debriefing results review, proofreading and final report.

**Results:**

The Brazilian Portuguese versions of the SMPI were well-accepted by respondents. Changes in about two-thirds of the items were considered necessary to obtain the final Brazilian Portuguese version of the SMPI.

**Conclusions:**

Both versions of the SMPI are now available in Brazilian Portuguese. The instrument could become an important option to enhance studies on melancholia in Portuguese-speaking samples.

## Introduction

The idea that depression is not a single syndrome but rather an entity that comprises several subtypes has been widely debated over the past few decades. Various views in this regard might be clustered around two main poles, namely, the unitary and the binary perspectives.^1 - 3^ According to the former, there is one single type of depression with variable intensity, with melancholic depression being the most severe one.^4^ The latter, i.e., the binary perspective, in turn, describes two main types of depression, namely: 1) reactive, neurotic or non-melancholic; and 2) endogenous, psychotic or melancholic.^1^

### Current issues on a melancholia specifier for major depression

The notion of major depressive disorder as described in the DSM essentially corresponds to the unitary perspective. The symptoms of melancholia are seen through a specifier for major depression. However, the items described in the DSM have been called into question for being unspecific and redundant vis-à-vis the larger notion of major depression.^5^ Also, DSM criteria are based on number of symptoms rather than on their intensity or quality, following the psychometric model instead of the clinimetric approach, in which major and minor symptoms of depression should be treated differently.^6 , 7^

Several authors have formulated specific scales to measure melancholia, the most significant of which are the Bech-Rafaelsen Melancholia Scale (MES),^8 , 9^ the Salpêtrière Retardation Rating Scale^10^ and the CORE System.^11^ The six-item Hamilton Depression Rating Scale (HAM-D6), developed by Bech et al.,^12^ has also been used to assess melancholia through a focus on depression-centered items. Caldieraro et al. found that the HAM-D6 did better than the 17-item version at identifying patients with melancholic depression according to the CORE.^13^

### The SMPI and the prototypical approach to melancholia

Parker et al.^1 , 14^ and Joyce et al.^15^ pointed to some limitations of the CORE and other traditional melancholia scales: psychomotor disturbance (PMD) signs are not so prevalent in younger patients and in those not at the worst episode stage; in addition, the CORE has an imprecise cutoff point. Thus, Parker et al. developed a new measure for the assessment of melancholia based on a prototypical scale, named the Sydney Melancholia Prototype Index (SMPI).^1^ This instrument combines psychomotor symptoms and clinical correlates,^1^ such as previous history and factors associated with clinical progression (as in the approach to the diagnosis of Parkinson’s disease).

The SMPI, an adaptation of the Self Report of Depressive Experiences (SERDEX), was formulated as a practical and all-encompassing inventory of depressive symptoms, including information on cognition, psychomotor retardation, previous personality and behavior. It is based on a prototypical model, thus differing from the classic scales (based on the intensity of symptoms), and is divided into two parts. Part one comprises two lists, each containing 12 features of depression, arranged in two columns (Description A and Description B). Description A, on the left, includes 12 features of melancholic depression, and Description B, on the right, 12 features of non-melancholic depression.^1^ Patients (self-report [SR] version) and psychiatrists (clinician-rated [CR] version) are requested to mark in both columns the items that best describe the experience of depression (whether current or historically). Some patients select items from the list of melancholic features only, others from the list of non-melancholic symptoms, and still others from both melancholic (Description A) and non-melancholic (Description B) symptoms. After this part, the instrument includes a generic question that requires patients (SMPI-SR) and psychiatrists (SMPI-CR) to indicate on a five-point scale which clinical description (Description A or B) best matches the patient’s symptoms.

The aim of the present study was to describe the cross-cultural adaptation of the SMPI by means of methods known to be efficient for this purpose.

## Methods

There are many methods to perform the cross-cultural adaptation of health assessment instruments. Based on a review of methods commonly used for translating and adapting instruments, in 2005 the International Society for Pharmacoeconomics and Outcomes Research (ISPOR) Task Force for Translation and Cultural Adaptation formulated a guideline with the steps required for self-report instruments.^16^

Translation of the SMPI to the Portuguese Brazilian language was based on the Principles of Good Practice, published by the ISPOR Task Force for Translation and Cultural Adaptation, which includes 10 steps described below.^16^

### 1. Preparation

Preparation comprises the following procedures: a) obtaining direct permission from the instrument developer for use and adaptation; b) inviting the instrument developer to be involved in the process; c) forming a research group to work in the target language, with the support of an external translator and the instrument developer. Requirements for the key in-country person are the following: being a native speaker of the target language and fluent in English, residing in the target country, and having knowledge and experience in this field and in research involving patients.

### 2. Forward translation

Translators should be selected who are native speakers of the target language and fluent in the source instrument language. Three translations are independently obtained in compliance with these criteria, which are then compared. The research group discusses the conceptual basis of the items and their context to avoid mistranslation and unclear terms.

### 3. Reconciliation

Discrepancies between the three independent translations are synthesized, resulting in a fourth version. Reconciliation might be performed by a) an independent native speaker; b) a group including the project manager and the forward translators; or c) the investigator charged with cognitive debriefing.

### 4. Back translation

This step provides quality control of the translated version. It is carried out by an external translator without previous contact with the original instrument or the forward translators.

### 5. Back translation review

This is one of the most important steps. The in-country project manager compares the back translated and source versions to check the equivalence of terms and solve discrepancies. The instrument developer is called to review the terms used to ensure the conceptual validity of the translated version.

### 6. Harmonization

Comparison of the back translation with back translations from other languages within the context of multicenter studies (not applicable to the SMPI).

### 7. Cognitive debriefing

The harmonized version in the target language is presented to a sample of in-country native speakers of the target language. A spreadsheet is used for each participant to assess each statement in the instrument. This step allows for: a) assessment of the level of comprehensibility and cognitive equivalence of the translation; b) testing translation alternatives suggested by the participants; c) highlighting any terms that might be inappropriate at a conceptual level; and d) identifying any other issues that cause confusion.

### 8. Review of cognitive debriefing results

This stage consists of discussion between the project manager and the research group to incorporate findings (more familiar terms or terms commonly used by respondents) of the debriefing process to improve the performance of the translation.

### 9. Proofreading

Final review, the aim of which is to check for minor errors that have been missed in the translation process.

### 10. Final report

The full process is described, including clear explanations of the reasons for all translation/wording choices made. This report is written by the project manager and should include a full description of the methods used and an item-by-item representation of all changes made.

This project was approved by the research ethics committee of Hospital de Clínicas de Porto Alegre (protocol 16-0540). All participants signed an informed consent form.

## Results

### Application of the ISPOR translation methodology

For the three first steps, permission for use and translation of the SMPI was directly obtained in the source country from its developer (Gordon Parker) by the project manager in Brazil. The research group in charge of translating and adapting the instrument to the Portuguese language was formed next, and Prof. Gordon Parker was invited to participate in the process. Then, three native Portuguese speakers fluent in English were selected to perform the forward translations, and three translations of the SMPI were independently obtained. The three independent forward translations were synthesized, resulting in a fourth version. Reconciliation was performed by a fourth translator, who is a native Portuguese speaker and had not participated in the step of forward translation. The colloquial style was preferred in all of these steps.

Next, an external and experienced translator performed the back translation of the SMPI into English. The selected translator did not have previous contact with the source instrument or the forward translators. The research group compared the back translation to the SMPI source version (in English) to check the equivalence of terms and solve discrepancies. The instrument developer reviewed the back translated terms to ensure their conceptual validity. For the cognitive debriefing, the SMPI-CR was presented to five physicians with experience in psychiatry (2nd- and 3rd-year residents), and the SPMI-SR version to five patients with depression under care at the psychiatric outpatient clinic and ward at Hospital de Clínicas de Porto Alegre. An ad hoc spreadsheet was used to assess the participants’ understanding of each statement in the scale. Procedures included: a) evaluation of the level of comprehensibility and cognitive equivalence of the translation; b) suggested translation alternatives; c) identification and recording of all items rated inappropriate at the conceptual level; and d) identification of items that caused confusion.

Finally, the research group discussed the results obtained in the cognitive debriefing step. The aim of this review was to incorporate findings (more familiar terms or terms commonly used by the respondents) from the debriefing process while keeping the meaning intended by the instrument developer. The final version was reviewed to check for minor errors that had been missed in the translation process, typos, grammatical errors and diacritical marks, among others, then a detailed report of the full process was prepared to facilitate the adaptation of the SMPI to different cultures.

**Figure 1:**
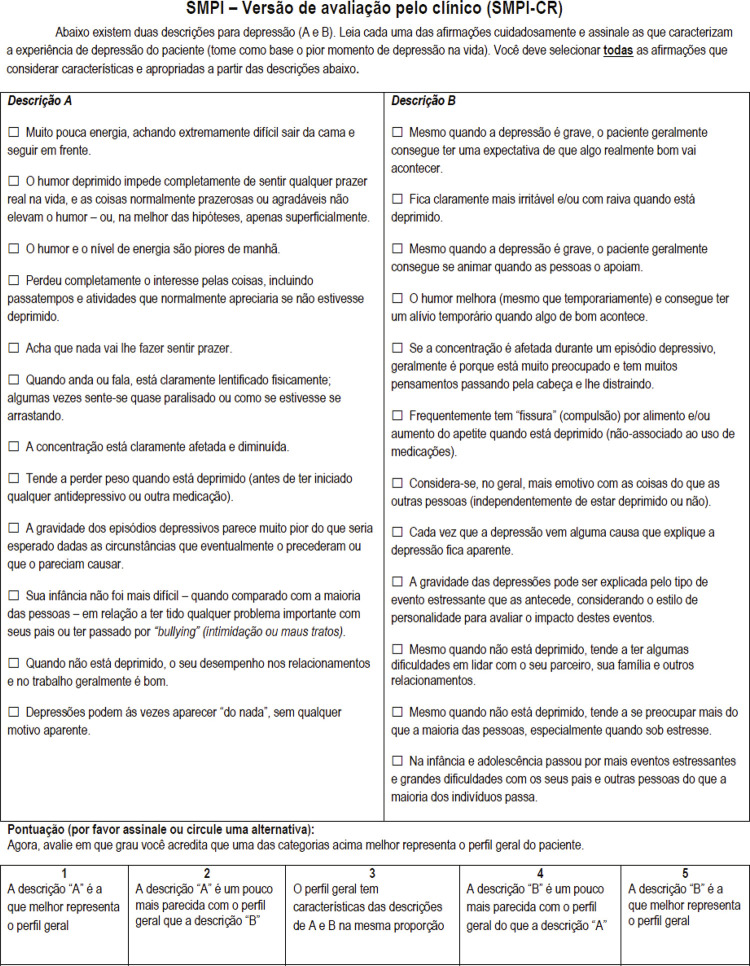
Final version of the Sydney Melancholia Prototype Index – Clinician Rated (SMPI-CR) in Brazilian Portuguese

**Figure 2:**
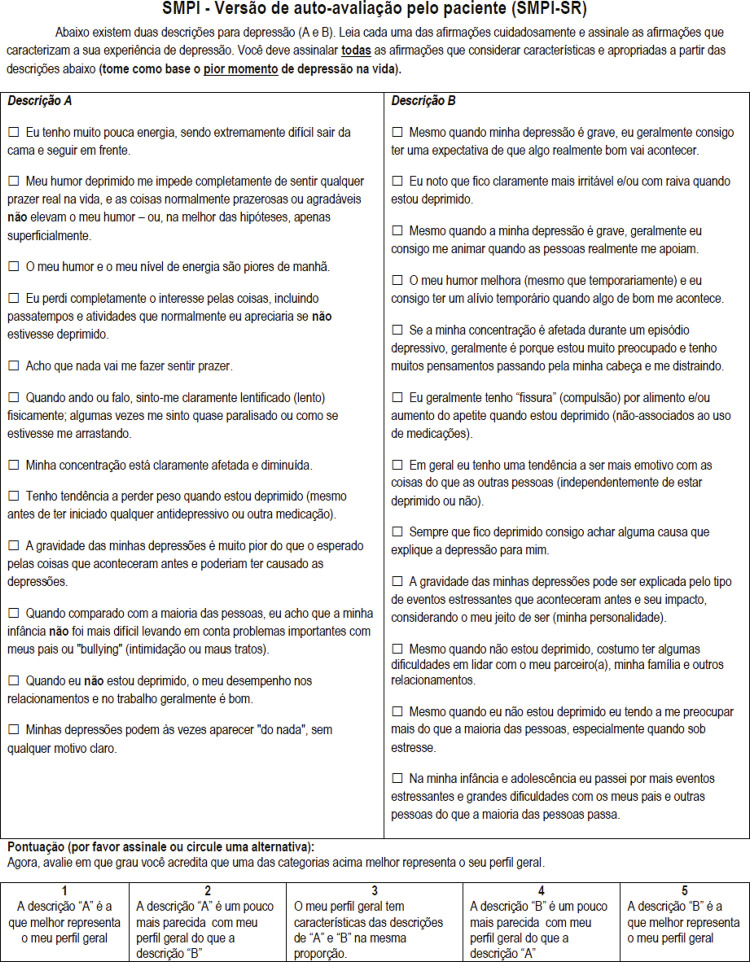
Final version of the Sydney Melancholia Prototype Index – Self-Report (SMPI-CR) in Brazilian Portuguese

Final versions in Portuguese of both SPMI-SR and SMPI-CR are shown in Figures 1 and 2 . Overall, cross-cultural adaptation of the SMPI required changes in about two-thirds of the items, including modifications in the internal order of statements and the incorporation of terms and expressions that were commonly used or colloquial in the Portuguese Brazilian language.

### Relevant changes made in specific items

Overall, the version presented was well-understood and accepted by the tested patients and physicians, except for five items in Description A and nine items in both Descriptions (A and B). Item #5 in Description A (“Can’t look forward to anything in life”) contains the phrasal verb “look forward,” which lacks a semantic equivalent in the Portuguese language. The instrument developer was thus consulted to develop an adequate translation. According to him, the core aspect of this item is to investigate “anhedonia relative to the future.” Therefore, the final version reads “ *acho que nada vai me fazer sentir prazer* ” in the SMPI-SR (“I think nothing will make me feel pleasure”) and “ *acha que nada vai lhe fazer sentir prazer* ” in the SMPI-CR (“The patient thinks nothing will make him or her feel pleasure”). Tables 1 and 2 summarize the relevant changes made in specific items during the process of translation. Tables S1 and S2, available as online-only supplementary material, present a full description of the process.

**Table 1:** Summary of results of the translation and adaptation of SMPI-SR items A5, A10, R1, R5, B6 and B9 into Brazilian Portuguese according to ISPOR recommendations

Original	Reconciliation and discussion results – changes when applied	Debriefing and discussion results – changes when applied	Final Brazilian Portuguese version
**Description A**	**-**	**-**	**Descrição A**
5- I find that I can’t look forward to anything in life.	I see that I cannot have any major expectations in life.	I think nothing will make me feel pleasure.	Acho que nada vai me fazer sentir prazer.
10- I don’t think that my early years were any more difficult – when compared to most people – in terms of having any major difficulties with parents or bullying.	-	I don’t think my childhood was more difficult – when compared to most people – regarding any major problems with parents or bullying (intimidation or mistreatment).	Quando comparado com a maioria das pessoas, eu acho que a minha infância não foi mais difícil levando em conta problemas importantes com meus pais ou “bullying” (intimidação ou maus tratos).
**RATING (please tick or circle one):**	**-**	**Punctuation (please check or circle one alternative):**	**Pontuação (por favor assinale ou circule uma alternativa):**
1- Description A best matches my overall profile.	-	Description “A” best represents my general profile.	A descrição “A” é a que melhor representa o meu perfil geral.
			
5- Description B best matches my overall profile.	-	Description “B” best represents my general profile.	A descrição “B” é a que melhor representa o meu perfil geral.
**Description B**	**-**	**Description B**	**Descrição B**
6- I often get (non-medication related) food cravings and/or increased appetite when I’m depressed.	I often have (non-drug related) food “cravings” (compulsive eating) and/or increased appetite when depressed.	I often have (non-drug related) food “cravings” (compulsion) and/or increased appetite when depressed.	Eu geralmente tenho “fissura” (compulsão) por alimento e/ou aumento do apetite quando estou deprimido (não-associados ao uso de medicações).
9- The severity of my depressions can be explained by the type of stressful events that precede them and the impact that these events have on me given my type of personality.	-	The severity of my depressions can be explained by the kind of stressful events that precede them and their impact given my personal style (my personality).	A gravidade das minhas depressões pode ser explicada pelo tipo de eventos estressantes que aconteceram antes e seu impacto, considerando o meu jeito de ser (minha personalidade).

ISPOR = International Society for Pharmacoeconomics and Outcomes Research; SMPI-SR = Sydney Melancholia Prototype Index – Self Report.

**Table 2:** - Summary of results of the translation and adaptation of SMPI-CR items A5, A10, R1, R5, B6 and B9 into Brazilian Portuguese according to ISPOR recommendations

Original	Reconciliation and discussion results – changes when applied	Debriefing and discussion results – changes when applied	Final Brazilian Portuguese version
**Description A**	**-**	**Description A**	**Descrição A**
5- Can’t look forward to anything in life.	Has no major expectations in life.	The patient thinks nothing will make him or her feel pleasure.	Acha que nada vai lhe fazer sentir prazer.
10- Early years were no more difficult – when compared to most people – in terms of having any major difficulties with parents or bullying.	-	Early years were no more difficult – when compared to most people – in terms of having any major difficulties with parents or bullying (intimidation or mistreatment).	Sua infância não foi mais difícil – quando comparado com a maioria das pessoas – em relação a ter tido qualquer problema importante com seus pais ou ter passado por “bullying” (intimidação ou maus tratos).
**RATING (please tick or circle one):**	**-**	**Punctuation (please check or circle one alternative):**	**Pontuação (por favor assinale ou circule uma alternativa):**
1- Description A best matches the overall profile.	-	Description ‘A’ is the one that best depicts the overall profile.	A descrição “A” é a que melhor representa o perfil geral.
5- Description B best matches the overall profile.	-	Description ‘B’ best depicts the overall profile.	A descrição “B” é a que melhor representa o perfil geral.
**Description B**	-	**Description B**	**Descrição B**
6- Often gets (non-medication related) food cravings and/or increased appetite when depressed.	Often has (non-drug related) food “cravings” (compulsive eating) and/or increased appetite when depressed.	Oftentimes has food ‘cravings’ (compulsion) and/or increased appetite when depressed (these events are non-drug-related).	Frequentemente tem “fissura” (compulsão) por alimento e/ou aumento do apetite quando está deprimido (não-associado ao uso de medicações).
9- The severity of depressions can be explained by the type of stressful events that precede them and their impact with personality style.	-	The severity of the depressions can be explained by the kind of stressful events that precede them, considering the personality style to evaluate the impact of these events.	A gravidade das depressões pode ser explicada pelo tipo de evento estressante que as antecede, considerando o estilo de personalidade para avaliar o impacto destes eventos.

ISPOR = International Society for Pharmacoeconomics and Outcomes Research; SMPI-CR = Sydney Melancholia Prototype Index – Clinician Report.

Item #9 in Description B, formulated in the SMPI-CR as “The severity of depressive episodes can be explained by the type of stressful events that precede them and their impact with personality style” was rated as too complex by both physicians and patients because it involved interpreting the relationship between the intensity of depressive episodes and potential triggers in addition to requiring temporal reasoning. Therefore, the final wording of item #9 in the SMPI-CR was changed to “The severity of depressive episodes can be explained by the type of stressful events that precede them, considering the personality style in the assessment of such events.” In the SMPI-SR, “my way of being” was used as a more colloquial expression for the term “personality,” which was kept, however, between brackets.

In the case of Description B, item #6, formulated in the SMPI-SR as “I often get (non-medication related) food cravings and/or increased appetite when I’m depressed,” and in the SMPI-CR as “Often gets (non-medication related) food cravings and/or increased appetite when depressed,” the order of the sentence was inverted, because that sounds more natural in the Portuguese language. The term “compulsion” was added between brackets to make the colloquial term “fissure” (craving) clearer.

In Description A, item #10 reads as follows in the SMPI-SR: “I don’t think that my early years were any more difficult – when compared to most people – in terms of having any major difficulties with parents or bullying”; and in the SMPI-CR: “Early years were no more difficult – when compared to most people – in terms of having any major difficulties with parents or bullying.” In these segments, the anglicism “bullying” was maintained because it is a commonly used word in Portuguese. Synonyms were added between brackets to make the meaning of this word clear.

Following the section devoted to prototypical characteristics of melancholic and non-melancholic depression, the SMPI includes a generic question to establish which prototypical set of clinical features best matches the patient’s condition. A single five-point response defines how well the patients match the melancholic or non-melancholic prototype. Subtle changes were made to items #1 and 5 to improve their understanding among Portuguese speakers.

## Discussion

The present article describes the translation and adaptation of the SMPI to the Brazilian Portuguese language in compliance with the guidelines formulated by the ISPOR Task Force for Translation and Cultural Adaptation. These guidelines allowed us to solve problems detected during the process of translation and cross-cultural adaptation of the SMPI. The review of the back translation by the instrument developer (Prof. Gordon Parker) contributed to us making the necessary changes to keep the translation faithful to his original intention in formulating each item in the scale. Cognitive debriefing, in turn, enabled the respondents (patients and professionals) to contribute to improving the translation.

Some problems inherent to translations from English to Latin languages were evident. For instance, the phrasal verb “look forward” has no exact equivalent in the Portuguese language. Therefore, careful selection was needed each time this expression appeared to maintain the meaning intended in the source version. Another aspect that deserves mention is use of the expressions “depressive symptoms” and “depressions” also to maintain the meaning intended by the instrument developer.

Overall, the Brazilian Portuguese version of the SMPI was accepted by the Brazilian patients. As a prototypical scale, patients can find in one of the two columns, each corresponding to a prototype, the symptoms that best match the description of their clinical features. As an additional apparent advantage, the SMPI requires patients to describe the features of the worst stage of their depressive experiences, which may not necessarily be representative of the present moment when they are responding to the instrument, a condition particularly relevant in the case of melancholia.

The SMPI has some limitations. Its original (prototypical) format may require additional explanations to those provided in the instructions. Additionally, the fact that the SMPI is not a quantitative, but rather a prototypical instrument, hinders its use for evaluating response to treatment, thus only allowing one to establish diagnostic categories (melancholic vs. non-melancholic depression).

Future studies are needed to evaluate not only the psychometric properties of the Brazilian Portuguese version of the SMPI but also and especially its clinical validity, which is an emerging concept in the clinimetric approach.^17 - 19^ This innovative clinically-based evaluation method combines the clinical judgment of experienced clinicians with item response theory (IRT) models for the assessment of measurement properties not restricted to the traditional psychometric model. Further researches to test the accuracy and concurrent validation of the SMPI are encouraged.

## Conclusion

The present study made the SMPI available in the Brazilian Portuguese language. This is one of the few instruments developed specifically for assessing melancholia. It comprises a clinician-rated and a patient self-report version, both of them now available in Portuguese.
