# Supplementary material for: Sydney Melancholia Prototype Index (SMPI): translation and cross-cultural adaptation to Brazilian Portuguese
Source: Trends Psychiatry Psychother. 2020 Oct 8;42(3):247–55. doi: 10.1590/2237-6089-2019-0045 (PMC7879070; doi:10.1590/2237-6089-2019-0045)
Supplement: Supplementary file 1 [file 2238-0019-trends-42-03-0247-suppl1.pdf]

Online-only supplementary material

Table S1 - Summary of results of the translation and adaptation of the SMPI-SR into Brazilian Portuguese according to ISPOR recommendations

Original	Reconciliation and discussion results – changes when applied	Debriefing and discussion results – changes when applied	Final Brazilian Portuguese version
**SMPI – Self-Report Questionnaire (SMPI-SR)**	**-**	**SMPI – Self-Administered Questionnaire (SMPI-SR)**	**SMPI – Versão de auto-avaliação pelo paciente (SMPI-SR)**
PATIENT ID	-	Patient Identification	Identificação do Paciente
Below are two descriptions of depression (A and B). Read each carefully and select any statement that characterizes your experience of depression (when feeling at your worst). You should select all appropriate and characteristic statements from the descriptions below.	-	There are two descriptions of depression below (A and B). Read each statement carefully and choose the ones that best describe your experience of depression. You must select **<u>all</u>** the statements that you consider typical and suitable from the descriptions below **(consider <u>the worst moment</u> of depression in life)**.	Abaixo existem duas descrições para depressão (A e B). Leia cada uma das afirmações cuidadosamente e assinale as afirmações que caracterizam a sua experiência de depressão. Você deve assinalar **<u>todas</u>** as afirmações que considerar características e apropriadas a partir das descrições abaixo **(tome como base o <u>pior momento</u> de depressão na vida)**.
**Description A**	-	-	**Descrição A**
1- I have very low energy and find it extremely hard to get out of bed and get going.	I have very low energy and find extremely difficulty in getting out of bed and starting the day.	I have very low energy and find extremely difficult getting out of bed and starting the day.	Eu tenho muito pouca energia, sendo extremamente difícil sair da cama e seguir em frente.
2- My depressed mood completely prevents me from getting any real pleasure in life, and normally pleasing or humorous things won't lift my mood – or, at best, only superficially.	-	-	Meu humor deprimido me impede completamente de sentir qualquer prazer real na vida, e as coisas normalmente prazerosas ou agradáveis não elevam o meu humor – ou, na melhor das hipóteses, apenas superficialmente.

3- My mood and energy levels are worse in the mornings.	-	-	O meu humor e o meu nível de energia são piores de manhã.
4- I completely lose interest in things, including hobbies and activities that I would usually enjoy when not depressed.	I completely lost interest in things, including hobbies and activities that I would usually value if not depressed.	-	Eu perdi completamente o interesse pelas coisas, incluindo passatempos e atividades que normalmente eu apreciaria se não estivesse deprimido.
5- I find that I can't look forward to anything in life.	I see that I cannot have any major expectations in life.	I think nothing will make me feel pleasure.	Acho que nada vai me fazer sentir prazer.
6- In walking and talking, I'm distinctly physically slowed, at times almost feeling 'paralysed' or as if I'm walking through sand.	-	When walking or talking, I am clearly physically slowed; sometimes, I feel almost paralysed or as if dragging a weight.	Quando ando ou falo, sinto-me claramente lentificado (lento) fisicamente; algumas vezes me sinto quase paralisado ou como se estivesse me arrastando.
7- My concentration is distinctly affected and slowed.	-	My concentration is clearly affected and diminished.	Minha concentração está claramente afetada e diminuída.
8- I tend to lose weight when I'm depressed (and before any antidepressant or other drugs are commenced).	-	I tend to lose weight when depressed (and before starting any treatment with antidepressants or other medication).	Tenho tendência a perder peso quando estou deprimido (mesmo antes de ter iniciado qualquer antidepressivo ou outra medicação).
9- The severity of my depressive episodes appears far worse than would be expected given the circumstances that may precede them or appear to cause them.	-	The severity of my depressive episodes seems much worse than would be expected considering the circumstances that might have preceded them or that seem to have caused them.	A gravidade das minhas depressões é muito pior do que o esperado pelas coisas que aconteceram antes e poderiam ter causado as depressões.
10- I don't think that my early years were any more difficult - when compared to most people - in terms of having any major difficulties with parents or bullying.	-	I don't think my childhood was more difficult – when compared to most people – regarding any major problems with parents or bullying (intimidation or mistreatment).	Quando comparado com a maioria das pessoas, eu acho que a minha infância não foi mais difícil levando em conta problemas importantes com meus pais ou "bullying" (intimidação ou maus tratos).

11- When I'm not depressed my relationships and work performance are generally good.	-	When I am not depressed, I show a generally good performance in relationships and work.	Quando eu não estou deprimido, o meu desempenho nos relacionamentos e no trabalho geralmente é bom.
	-		
12- My depressions can sometimes come 'out of the blue' without any particularly clear reason.	-	My depressive symptoms can sometimes appear "out of the blue", for no apparent reason.	Minhas depressões podem às vezes aparecer "do nada", sem qualquer motivo claro.
**RATING: (please tick or circle one):**	-	**Punctuation (please check or circle one alternative):**	**Pontuação (por favor assinale ou circule uma alternativa):**
Now, rate the degree to which you believe one of the categories above best matches your overall profile.	-	Now, evaluate to what extent you believe that one of the categories above best represents your general profile.	Agora, avalie em que grau você acredita que uma das categorias acima melhor representa o seu perfil geral.
1- Description A best matches my overall profile.	-	Description "A" best represents my general profile.	A descrição "A" é a que melhor representa o meu perfil geral.
2- Description A is somewhat closer to my overall profile. than Description B.	-	Description "A" is somewhat closer to my general profile than description "B".	A descrição "A" é um pouco mais parecida com meu perfil geral do que a descrição "B".
3- My overall profile has equal features of Descriptions A and B.	-	My general profile has characteristics of descriptions A and B on the same proportion.	O meu perfil geral tem características das descrições de "A" e "B" na mesma proporção.
4- Description B is somewhat closer to my overall profile than Description A.	-	Description "B" is somewhat closer to my general profile than description "A".	A descrição "B" é um pouco mais parecida com o meu perfil geral do que a descrição "A".
5- Description B best matches my overall profile.	-	Description "B" best represents my general profile.	A descrição "B" é a que melhor representa o meu perfil geral.

**Description B**	**-**	**-**	**Descrição B**
1- Even when my depression is severe, I can generally look forward to something really nice coming up.	-	Even when depression is severe, I can generally expect that something really nice might happen.	Mesmo quando minha depressão é grave, eu geralmente consigo ter uma expectativa de que algo realmente bom vai acontecer.
2- I find that I become distinctly more irritable and/or angry when I'm depressed.	-	I clearly notice that I become more irritable and/or angry when depressed.	Eu noto que fico claramente mais irritável e/ou com raiva quando estou deprimido.
3- Even when my depression is severe, I can generally be cheered up when people are really supportive.	-	Even when my depression is severe, I can generally be cheered up when people really support me.	Mesmo quando a minha depressão é grave, geralmente eu consigo me animar quando as pessoas realmente me apoiam.
4- My mood lifts (even if temporarily) and I can obtain some temporary relief when something nice happens.	-	My mood lifts (even if temporarily) and I can feel a temporary relief when something good happens.	O meu humor melhora (mesmo que temporariamente) e eu consigo ter um alívio temporário quando algo de bom me acontece.
5- If my concentration is affected during a depressive episode, it is usually because I am worrying too much and have lots of thoughts going through my head distracting me.	-	If my concentration is affected during a depressive episode, it is usually because I am too worried and have lot of distracting thoughts going through my head.	Se a minha concentração é afetada durante um episódio depressivo, geralmente é porque estou muito preocupado e tenho muitos pensamentos passando pela minha cabeça e me distraindo.
6- I often get (non-medication related) food cravings and/or increased appetite when I'm depressed.	I often have (non-drug related) food "cravings" (compulsive eating) and/or increased appetite when depressed.	I often have (non-drug related) food "cravings" (compulsion) and/or increased appetite when depressed.	Eu geralmente tenho "fissura" (compulsão) por alimento e/ou aumento do apetite quando estou deprimido (não-associados ao uso de medicações).
7- I view myself as generally more inclined than most people to become emotional about things (regardless of whether I'm depressed or not).	I consider myself more likely to feel emotional about things (regardless of being depressed or not) in general.	I consider myself more likely to feel emotional about things (regardless of being depressed or not).	Em geral eu tenho uma tendência a ser mais emotivo com as coisas do que as outras pessoas (independentemente de estar deprimido ou não).

8- Every time I get depressed, I can find some cause that explains the depression to me.	-	Whenever I become depressed I can pinpoint some cause that explains depression.	Sempre que fico deprimido consigo achar alguma causa que explique a depressão para mim.
9- The severity of my depressions can be explained by the type of stressful events that precede them and the impact that these events have on me given my type of personality.	-	The severity of my depressions can be explained by the kind of stressful events that precede them and their impact given my personality style (my personality).	A gravidade das minhas depressões pode ser explicada pelo tipo de eventos estressantes que aconteceram antes e seu impacto, considerando o meu jeito de ser (minha personalidade).
10- Even when I'm not depressed, I tend to have some difficulties in dealing with my partner, family and other relationships.	-	Even when I am not depressed, I tend to have some difficulties in dealing with partners, family and other relationships.	Mesmo quando não estou deprimido, costumo ter algumas dificuldades em lidar com o meu parceiro(a), minha família e outros relacionamentos.
11- Even when I'm not depressed, I tend to worry more than most people, particularly when under stress.	-	Even when I am not depressed, I tend to worry more than most people, especially when under stress.	Mesmo quando eu não estou deprimido eu tendo a me preocupar mais do que a maioria das pessoas, especialmente quando sob estresse.
12- In childhood and adolescence, I experienced more stressful events and major difficulties with my parents and others than most people experience.	-	In my childhood and adolescence, I experienced more stressful events and major difficulties with my parents than most people do.	Na minha infância e adolescência eu passei por mais eventos estressantes e grandes dificuldades com os meus pais e outras pessoas do que a maioria das pessoas passa.

ISPOR = International Society for Pharmacoeconomics and Outcomes Research; SMPI-SR = Sydney Melancholia Prototype Index – Self Report.

Table S2 - Summary of results of the translation and adaptation of Sydney Melancholia Prototype Index – Clinician-Rated version (SMPI-CR) into Brazilian Portuguese according to ISPOR recommendations

Original	Reconciliation and discussion results – changes when applied	Debriefing and discussion results – changes when applied	Final Brazilian Portuguese version
**SMPI – Clinician-Rated version (SMPI-CR)**	**-**	**SMPI – Practitioner evaluation version (SMPI-CR)**	**SMPI – Versão de avaliação pelo clínico (SMPI-CR)**
PATIENT ID	-	Patient Identification	Identificação do Paciente
Below are two descriptions of depression (A and B). Read each carefully and select any statement that characterises the patient's experience of depression (when feeling at their worst).<br>You should select all appropriate and characteristic statements from the descriptions below.	-	There are two descriptions of depression below (A and B). Read each statement carefully and choose the ones that best describe the patient's experience of depression (consider the **worst moment** of or depression in life). You must select **all** the statements that you consider typical and suitable from the descriptions below.	Abaixo existem duas descrições para depressão (A e B). Leia cada uma das afirmações cuidadosamente e assinale as que caracterizam a experiência de depressão do paciente (tome como base o **pior momento** de depressão na vida). Você deve selecionar **todas** as afirmações que considerar características e apropriadas a partir das descrições abaixo.
**Description A**	**-**	**Description A**	**Descrição A**
1- Very low energy and finding it extremely hard to get out of bed and get going.	Very low energy and finds extremely difficulty in getting out of bed and starting the day.	Very low energy, finding it extremely hard to get out of bed and move on.	Muito pouca energia, achando extremamente difícil sair da cama e seguir em frente.
2- Depressed mood completely prevents patient getting any real pleasure in life, and normally pleasing or humorous things won't lift mood – and at best, only superficially.	-	-	O humor deprimido impede completamente de sentir qualquer prazer real na vida, e as coisas normalmente prazerosas ou agradáveis não elevam o humor – ou, na melhor das hipóteses, apenas superficialmente.
3- Mood and energy levels are worse in the mornings.	-	-	O humor e o nível de energia são piores de manhã.

4- Completely loses interest in things, including hobbies and activities that would usually be enjoyed when not depressed.	Completely lost interest in things, including hobbies and activities that would usually enjoy/find pleasurable if not depressed.	The patient has completely lost interest in things, including hobbies and activities that he or she would usually value if not depressed.	Perdeu completamente o interesse pelas coisas, incluindo passatempos e atividades que normalmente apreciaria se não estivesse deprimido.
5- Can't look forward to anything in life.	Has no major expectations in life.	The patient thinks nothing will make him or her feel pleasure.	Acha que nada vai lhe fazer sentir prazer.
6- In walking and talking, is distinctly physically slowed, at times almost feeling 'paralysed' or as if walking through sand.	-	-	Quando anda ou fala, está claramente lentificado fisicamente; algumas vezes sente-se quase paralisado ou como se estivesse se arrastando.
7- Concentration is distinctly affected and slowed.	-	-	A concentração está claramente afetada e diminuída.
8- Tends to lose weight when depressed (and before any antidepressant or other drugs are commenced).	-	Tends to lose weight when depressed (before starting any treatment with antidepressants or other medication).	Tende a perder peso quando está deprimido (antes de ter iniciado qualquer antidepressivo ou outra medicação).
9- The severity of depressive episodes appears far worse than would be expected given the circumstances that may precede them or appear to cause them.	-	The severity of the depressive episodes seems much worse than would be expected considering the circumstances that might have preceded them or that seem to have caused them.	A gravidade dos episódios depressivos parece muito pior do que seria esperado dadas as circunstâncias que eventualmente o precederam ou que o pareciam causar.
10- Early years were no more difficult - when compared to most people - in terms of having any major difficulties with parents or bullying.	-	Early years were no more difficult - when compared to most people - in terms of having any major difficulties with parents or bullying (intimidation or mistreatment).	Sua infância não foi mais difícil - quando comparado com a maioria das pessoas - em relação a ter tido qualquer problema importante com seus pais ou ter passado por "bullying" (intimidação ou maus tratos).
11- When not depressed, relationships and work performance are generally good.	-	-	Quando não está deprimido, o seu desempenho nos relacionamentos e no trabalho geralmente é bom.

12- Depressions can sometimes come ‘out of the blue’ without any particularly clear reason.	-	-	Depressões podem ás vezes aparecer "do nada", sem qualquer motivo aparente.
**RATING (please tick or circle one):**	-	**Punctuation (please check or circle one alternative):**	**Pontuação (por favor assinale ou circule uma alternativa):**
Now, rate the degree to which you believe one of the categories above best matches the patient’s overall profile.	-	Now, evaluate to what extent you believe that one of the categories above best represents the overall profile of the patient.	Agora, avalie em que grau você acredita que uma das categorias acima melhor representa o perfil geral do paciente.
1- Description A best matches the overall profile.	-	Description ‘A’ is the one that best depicts the overall profile.	A descrição “A” é a que melhor representa o perfil geral.
2- Description A is somewhat closer to the overall profile than Description B.	-	-	A descrição “A” é um pouco mais parecida com o perfil geral que a descrição “B”.
3- The overall profile has equal features of Descriptions A and B.	-	-	O perfil geral tem características das descrições de A e B na mesma proporção.
4- Description B is somewhat closer to the overall profile than Description A.	-	-	A descrição “B” é um pouco mais parecida com o perfil geral do que a descrição “A”.
5- Description B best matches the overall profile.	-	Description ‘B’ best depicts the overall profile.	A descrição “B” é a que melhor representa o perfil geral.
**Description B**	-	**Description B**	**Descrição B**
1- Even when depression is severe, the patient can generally look forward to something really nice coming up.	-	Even when depression is severe, the patient can generally expect that something really good might happen.	Mesmo quando a depressão é grave, o paciente geralmente consegue ter uma expectativa de que algo realmente bom vai acontecer.
2- Becomes distinctly more irritable and/or angry when depressed.	-	-	Fica claramente mais irritável e/ou com raiva quando está deprimido.

3- Even when depression is severe, the patient can generally be cheered up when people are really supportive.		Even when depression is severe, the patient can generally be cheered up when people support him or her.	Mesmo quando a depressão é grave, o paciente geralmente consegue se animar quando as pessoas o apoiam.
4- Mood lifts (even if temporarily) and can obtain some temporary relief when something nice happens.	-	Mood lifts (even if temporarily) and the patient can feel a temporary relief when something good happens.	O humor melhora (mesmo que temporariamente) e consegue ter um alívio temporário quando algo de bom acontece.
5- If concentration is affected during a depressive episode, it is usually because of worrying too much and having lots of distracting thoughts.	-	-	Se a concentração é afetada durante um episódio depressivo, geralmente é porque está muito preocupado e tem muitos pensamentos passando pela cabeça e lhe distraindo.
6- Often gets (non-medication related) food cravings and/or increased appetite when depressed.	Often has (non-drug related) food "cravings" (compulsive eating) and/or increased appetite when depressed.	Oftentimes has food ‘cravings’ (compulsion) and/or increased appetite when depressed (these events are non-drug-related).	Frequentemente tem “fissura” (compulsão) por alimento e/ou aumento do apetite quando está deprimido (não-associado ao uso de medicações).
7- Views self as generally more inclined than most people to become emotional about things (regardless of whether depressed or not).	Considers himself or herself more likely to feel emotional about things (regardless of being depressed or not) in general.	Considers himself or herself more likely to feel emotional about things than other people (regardless of being depressed or not).	Considera-se, no geral, mais emotivo com as coisas do que as outras pessoas (independentemente de estar deprimido ou não).
8- Every time depression develops, some cause that explains the depression is apparent.	-	-	Cada vez que a depressão vem alguma causa que explique a depressão fica aparente.
9- The severity of depressions can be explained by the type of stressful events that precede them and their impact with personality style.	-	The severity of the depressions can be explained by the kind of stressful events that precede them, considering the personality style to evaluate the impact of these events.	A gravidade das depressões pode ser explicada pelo tipo de evento estressante que as antecede, considerando o estilo de personalidade para avaliar o impacto destes eventos.
10- Even when not depressed, tends to have some difficulties in dealing with their partner, family and other relationships.	-	-	Mesmo quando não está deprimido, tende a ter algumas dificuldades em lidar com o seu parceiro, sua família e outros relacionamentos.

11- Even when not depressed, patient tends to worry more than most people, particularly when under stress	-	-	Mesmo quando não está deprimido, tende a se preocupar mais do que a maioria das pessoas, especialmente quando sob estresse.
12- In childhood and adolescence, the patient experienced more stressful events and major difficulties with parents and others than most people experience.	-	In childhood and adolescence, the patient experienced more stressful events and major difficulties with his or her parents than most people do.	Na infância e adolescência passou por mais eventos estressantes e grandes dificuldades com os seus pais e outras pessoas do que a maioria dos indivíduos passa.

ISPOR = International Society for Pharmacoeconomics and Outcomes Research; SMPI-CR = Sydney Melancholia Prototype Index – Clinician Rated.
